# JAK-STAT signaling: a key mechanism of immune microenvironment remodeling in breast cancer and novel strategies for targeted therapy

**DOI:** 10.3389/fonc.2026.1947188

**Published:** 2026-10-20

**Authors:** Junfang Qiao, Tingting Sun, Guoqiang Wang, Ying Yan, Weibing Tong

**Affiliations:** 1 Graduate School of Inner Mongolia Medical University, Hohhot, China; 2 Breast Tumor Center, Beijing University Cancer Hospital Inner Mongolia Hospital, Hohhot, China; 3 Key Laboratory of Carcinogenesis and Translational Research (Ministry of Education), Department of Breast Oncology, Peking University Cancer Hospital and Institute, Beijing, China

**Keywords:** breast cancer, combination therapy, immune evasion, JAK-STAT signaling pathway, targeted therapy, tumor immune microenvironment

## Abstract

The JAK-STAT signaling pathway serves as a critical hub for cytokine signal transduction, and its aberrant activation plays a decisive role in shaping the immune microenvironment of breast cancer. This review systematically elucidates how this pathway contributes to the construction of an immunosuppressive microenvironment through upregulating PD-L1 expression, recruiting and activating myeloid-derived suppressor cells (MDSCs), promoting regulatory T cell (Treg) generation, while concomitantly suppressing the functions of CD8+ T cells and natural killer (NK) cells. Although targeted therapy against this pathway, particularly in combination with immune checkpoint inhibitors, has demonstrated promising potential, several challenges remain, including pathway compensation, low inhibitor selectivity, associated toxicities, and the lack of predictive biomarkers. Future directions should focus on the development of highly selective STAT inhibitors, exploration of immunophenotype-guided combination strategies, establishment of robust biomarker systems, and in-depth investigation of the dynamic interplay between this pathway and the immune microenvironment, thereby providing a theoretical foundation for combined immunotherapy in breast cancer.

## 1 Introduction

Breast cancer (BC) is a highly prevalent malignancy among women worldwide, with persistently elevated incidence and mortality rates, posing a serious threat to women’s health (1, 2). Breast cancer encompasses several subtypes, including Luminal, HER2-positive, and triple-negative breast cancer, each of which corresponds to distinct optimal treatment strategies (3). Currently, the primary treatment modalities for breast cancer include radiotherapy, chemotherapy, surgery, and endocrine therapy (4). However, challenges such as high rates of recurrence, metastasis, and drug resistance persist. Therefore, elucidating the molecular mechanisms underlying these processes is essential for developing effective therapeutic strategies for breast cancer (5). The Janus kinase (JAK)/signal transducer and activator of transcription (STAT) pathway, composed of JAKs, JAK-associated receptors, and STATs (6), serves as a critical signaling cascade for cytokines, growth factors, and hormones (7). This pathway not only regulates cell proliferation, apoptosis, and metastasis but also plays a profound role in immune modulation and inflammatory responses (8). Recent studies have demonstrated that the JAK/STAT signaling pathway plays a critical role in regulating the tumor immune microenvironment, influencing immune cell infiltration, differentiation, and functional status, and is closely associated with the expression of immune checkpoints. Therefore, this review focuses on the regulatory mechanisms of the JAK/STAT signaling pathway in the breast cancer immune microenvironment and its potential implications for immunotherapy, aiming to provide a theoretical reference for the development of combined immunotherapeutic strategies for breast cancer.

## 2 Composition and working mechanism of the JAK/STAT pathway

The JAK/STAT signaling pathway serves as a critical hub mediating the actions of various cytokines and growth factors (9),directly transducing signals from transmembrane receptors to the nucleus, and plays a fundamental role in the regulation of the tumor immune microenvironment (10). JAKs are non-transmembrane tyrosine kinases comprising four members: JAK1, JAK2, JAK3, and TYK2. STATs are a family of cytoplasmic target proteins located downstream of JAKs, consisting of seven members: STAT1, STAT2, STAT3, STAT4, STAT5a, STAT5b, and STAT6 (11). Activation of the canonical JAK–STAT signaling pathway is initiated by cytokine ligand binding to the extracellular domain of the cognate receptor. This binding induces receptor dimerization, which in turn brings JAK kinases that are constitutively associated with the receptor intracellular domains into close proximity, leading to activation of their kinase domains via trans-phosphorylation (12). Activated JAKs then phosphorylate specific tyrosine residues within the receptor intracellular domain, thereby generating docking sites for STAT proteins (13). Cytoplasmic STAT proteins in a latent state use their SH2 domains to recognize and dock onto these phosphorylated sites and are subsequently phosphorylated by JAKs at conserved C-terminal tyrosine residues (e.g., Tyr705 of STAT3 and Tyr701 of STAT1) (14). Phosphorylated STAT proteins form homodimers or heterodimers through reciprocal SH2–phosphotyrosine interactions and enter the nucleus via an importin-α/β complex–mediated nuclear transport mechanism (15). In the nucleus, STAT dimers specifically recognize and directly bind to GAS elements in the promoter regions of target genes. Through their transactivation domains, they recruit coactivators, thereby initiating the transcriptional program of target genes (16). The JAK-STAT signaling pathway is a dynamic equilibrium system subject to tight regulation; its overactivation can disrupt immune homeostasis, triggering aberrant immune responses and internal milieu imbalance, which in turn lead to inflammation and tissue damage (17). Negative regulation of the JAK-STAT pathway primarily involves the following three families of negative regulators: suppressors of cytokine signaling (SOCS/CIS), protein inhibitors of activated STAT (PIAS), and protein tyrosine phosphatases (PTPs) (18). These regulatory factors are capable of suppressing JAK-STAT signaling activation and play a critical role in maintaining immune homeostasis; their deficiency can lead to persistent activation of this pathway, thereby promoting tumor proliferation and progression. However, individual members of the STAT family assume distinctly different functional roles in immune regulation. As a key downstream mediator of type I/II interferon signaling pathways, activated STAT1 promotes the upregulation of MHC class I molecules, the release of T cell chemokines such as CXCL9/10, and the enhancement of cytotoxic T cell and NK cell killing activity, thereby playing a central role in antitumor immune surveillance (19–21). In breast cancer, however, STAT3 and STAT5 predominantly mediate immunosuppressive effects: STAT3 directly drives the transcriptional expression of PD-L1 and promotes the accumulation and expansion of myeloid-derived suppressor cells (MDSCs) and regulatory T cells (Tregs) within the tumor microenvironment (22). STAT5, in turn, promotes Treg differentiation and maintains their suppressive function by upregulating FOXP3 expression (23). A notable mechanism is that STAT1 and STAT3 can form heterodimers in the cytoplasm and, after co-translocating into the nucleus, bind to the PD-L1 promoter region to maintain its sustained expression; blocking STAT1 or STAT3 alone only leads to partial downregulation of PD-L1, whereas combined inhibition of both is required to achieve complete suppression, suggesting a functional antagonistic relationship between the two (24). Furthermore, the ShcA adaptor protein pathway concurrently enhances STAT3-mediated immunosuppressive signaling and attenuates STAT1-driven immune surveillance, thereby synergistically promoting immune evasion in breast cancer (25).

## 3 Roles of the JAK-STAT pathway in other cancers

Aberrant activation of the JAK-STAT signaling pathway is commonly observed in various malignancies, with its activation patterns and immunomodulatory effects exhibiting notable heterogeneity across different tumor types. A systematic synthesis of these differences may facilitate a deeper understanding of the commonalities and specificities of the JAK–STAT pathway’s modes of action in breast cancer, thereby providing a reference framework for mechanistic exploration and the formulation of targeted intervention strategies. In myeloproliferative neoplasms (MPN), mutations such as JAK2-V617F at specific loci drive persistent activation of this pathway, which not only promotes malignant expansion of hematopoietic cells but also exerts profound effects on immune cell differentiation and function, collectively contributing to the formation of an aberrant immune microenvironment (26–28). In response to this phenomenon, JAK inhibitors (e.g., ruxolitinib) have been widely applied in clinical practice (29). Meanwhile, the immunomodulatory agent IFNα2b can restore T-cell immune function by suppressing the STAT3-FGL2 axis (30). Furthermore, the efficacy of PD-1 inhibitor monotherapy remains limited, and clinical studies investigating combination regimens, such as fedratinib combined with nivolumab, are currently underway (31), offering novel strategies to overcome immune tolerance. MPNs provide a typical example of “mutation-driven” constitutive activation of the JAK–STAT pathway, and the clinical success of JAK inhibitors in MPNs offers an important reference for targeting this pathway in breast cancer; however, the mutation-driven activation pattern in MPNs is fundamentally different from the signal-dependent activation in breast cancer, suggesting that directly adopting therapeutic strategies established in MPNs may fail to achieve the expected outcomes in breast cancer. In hepatocellular carcinoma (HCC), activation of this pathway does not arise from direct mutations but rather serves as a critical bridge connecting chronic inflammation to malignant transformation: persistent activation of the IL-6/JAK/STAT3 axis drives tumor progression through a dual mechanism, including direct upregulation of genes such as the transcription factor c-Myc and Cyclin D1 to promote aberrant hepatocyte proliferation and survival (32),as well as indirect induction of epithelial-mesenchymal transition (EMT) to enhance tumor cell invasion and metastatic capacity, while also activating hepatic stellate cells to promote liver fibrosis/cirrhosis and an immunosuppressive microenvironment (33). Mechanistically, STAT3 activation upregulates PD-L1 expression to induce immune evasion and promotes the expansion of myeloid-derived suppressor cells (MDSCs) to mediate immunosuppression (34). Immunotherapeutic strategies targeting STAT3 are currently under investigation, and combination regimens such as TLR9 agonists with PD-1 inhibitors have demonstrated synergistic antitumor effects in HCC (35). HCC and breast cancer (particularly TNBC) exhibit striking similarities in their modes of JAK–STAT pathway activation, both manifesting cytokine-driven, signal-dependent activation and leveraging STAT3 to upregulate PD-L1 and recruit MDSCs to establish an immunosuppressive microenvironment. Therefore, combination therapeutic strategies targeting the IL-6/JAK/STAT3 axis in HCC may provide directly transferable insights for breast cancer. In head and neck squamous cell carcinoma (HNSCC) and lung cancer, persistent STAT3 activation serves as a core mechanism driving immune evasion (36). On one hand, it directly upregulates programmed death-ligand 1 (PD-L1) expression, leading to T-cell dysfunction (37);on the other hand, it modulates the functions of myeloid-derived suppressor cells (MDSCs) and regulatory T cells (Tregs) to establish a potent immunosuppressive microenvironment (38). The mechanisms by which STAT3 drives immune evasion in HNSCC and lung cancer are highly consistent with those in breast cancer. Preclinical evidence from these cancer types demonstrating that JAK/STAT inhibitors downregulate PD-L1 expression and sensitize tumors to PD-1/PD-L1 blockade may provide directly applicable empirical support for the design of combination immunotherapeutic strategies in breast cancer. Collectively, the JAK-STAT pathway plays distinct immunomodulatory roles across different tumor types: in MPN, it manifests as genetically driven immune homeostasis dysregulation; in HCC, it serves as a hub for inflammation-driven immune remodeling; and in HNSCC and lung cancer, it acts as a dominant driver of immune evasion. Compared with the aforementioned tumors, activation of the JAK-STAT pathway in breast cancer exhibits unique immunoregulatory complexity. Through this cross-cancer comparison, it becomes clear that the activation mode of the JAK–STAT pathway in breast cancer (signal-dependent, rarely mutation-driven) and its immunoregulatory functions (PD-L1 upregulation, MDSC/Treg recruitment) share greater commonality with HCC, HNSCC, and lung cancer, while being fundamentally distinct from MPN.

## 4 Aberrant activation of the JAK-STAT pathway in breast cancer

In breast cancer progression, dysregulation of the JAK-STAT pathway is a critical factor driving tumor malignancy. This aberrant activation typically does not arise from a single cause but results from the combined effects of three aspects: sustained stimulation by upstream signals, alterations in the pathway components themselves, and failure of the intrinsic inhibitory mechanisms within the pathway. This process profoundly influences the shaping of the tumor immune microenvironment. Furthermore, the activation patterns and immunomodulatory functions of this pathway exhibit significant heterogeneity across different molecular subtypes of breast cancer, displaying distinct immunophenotypic characteristics.

### 4.1 Molecular mechanisms of aberrant activation

The breast cancer tumor microenvironment sustains activation of the JAK-STAT signaling pathway through various cytokines, thereby influencing immune regulation and PD-L1 expression. Among these, IL-6 is aberrantly overexpressed in triple-negative breast cancer (TNBC) and activates the JAK-STAT3 pathway, forming a self-amplifying signaling loop that directly contributes to the transcriptional upregulation of PD-L1 (39). Leukemia inhibitory factor (LIF) maintains cancer stem cell properties through the same pathway (40),while granulocyte-macrophage colony-stimulating factor (GM-CSF) modulates immune cell functions via the JAK2-STAT5 pathway, collectively contributing to the formation of an immunosuppressive microenvironment (41). Extensive crosstalk exists between the JAK-STAT pathway and growth factor signaling pathways. In HER2-positive breast cancer, epidermal growth factor (EGF) and fibroblast growth factor (FGF) can activate STAT through their receptor tyrosine kinases, and this mechanism has been associated with resistance to targeted therapy (42). In Luminal breast cancer, prolactin activates the JAK2-STAT5a signaling pathway and synergizes with estrogen receptor signaling to regulate the expression of immune-related genes (43). Molecular alterations in pathway components also affect immune regulation. Gene amplification of STAT3 and STAT5 can enhance pathway activity (44),while post-translational modifications of STAT3, such as Ser727 phosphorylation and Lys685 acetylation, can prolong its transcriptional activity (45), which may affect the expression of immune-related genes, including PD-L1. Silencing of SOCS family proteins (particularly SOCS1 and SOCS3) due to promoter hypermethylation attenuates the negative regulation of JAK-STAT signaling, a phenomenon particularly common in TNBC (46), and directly affects the tumor immune tolerance status. Based on the aforementioned mechanisms, immunotherapeutic strategies targeting the JAK-STAT pathway are rapidly evolving: preclinical studies have demonstrated that IL-6R monoclonal antibody combined with PD-L1 antibody synergistically inhibits TNBC progression, and sequential immunotherapy (PD-L1 followed by IL-6R) achieves optimal survival benefits (47).

### 4.2 Differential immunomodulatory patterns across molecular subtypes

The differences in JAK/STAT pathway activation patterns across breast cancer subtypes arise from the diversity of their upstream driving signals: IL-6/IL-6R activates STAT3 through JAK1/JAK2; IFN-γ primarily activates STAT1 through JAK1/JAK2; GM-CSF activates STAT5 through JAK2; prolactin activates STAT5a through JAK2; Receptor Tyrosine Kinases(RTKs) such as HER2 activate STAT3 through Src kinase(SRC) and JAK2; and TYK2 primarily mediates type I IFN (IFNα/β) signaling. These differences determine the functional positioning and therapeutic strategies of the JAK/STAT pathway in each subtype (48).

In TNBC, IL-6 activates JAK1/JAK2 through IL-6R, which in turn phosphorylates STAT3,the IL-6/JAK2/STAT3 axis promotes tumor progression by maintaining cancer stem cell properties and enhancing invasive and metastatic capacity (Tumor cell-intrinsic effects), while concomitantly upregulating PD-L1 expression and recruiting MDSCs(Immunomodulatory effects on the tumor microenvironment), thereby establishing an immunosuppressive microenvironment (49). Mechanistic studies have revealed that autocrine IL-6 signaling upregulates PD-L1 expression through the JAK2/STAT3 pathway(see Section 4.4 for specific molecular mechanisms). The immune microenvironment of TNBC is not uniform; some tumors exhibit an immune-excluded or immune-desert phenotype rather than an immune-infiltrated one. In immune-infiltrated TNBC, the core positioning of JAK–STAT-targeted therapy is to sensitize pre-existing antitumor immune responses, whereas after the emergence of immunotherapy resistance, its positioning shifts to overcoming acquired resistance (50). In HER2-positive breast cancer, HER2 homodimers and heterodimers can directly activate STAT3 through SRC kinase and JAK2, forming an alternative survival signaling axis independent of the phosphatidylinositol 3-kinase (PI3K)/AKT and mitogen-activated protein kinase (MAPK) pathways (51). NCAPG condensins mediate trastuzumab resistance by phosphorylating SRC and enhancing STAT3 nuclear localization (52); the PDGFRA receptor tyrosine kinase/STAT3/IL-6 axis is also implicated in resistance, and combined treatment with PDGFRA inhibitors and anti-HER2 therapy can suppress the activity of this axis (53). This mechanism is not only associated with trastuzumab resistance but also influences the regulation of tumor immune responses (54). The degree of immune infiltration in HER2+ breast cancer typically lies between that of TNBC and Luminal breast cancer, with detectable but substantially less abundant T cell infiltration than in TNBC. Therefore, the core positioning of JAK–STAT-targeted therapy in this subtype is not to sensitize immune responses, but rather to overcome acquired resistance to trastuzumab(Tumor cell-intrinsic effects) and to improve the immunosuppressive microenvironment by inhibiting STAT3-mediated PD-L1 expression(Immunomodulatory effects on the tumor microenvironment) (55). In Luminal breast cancer, the function of the JAK-STAT pathway exhibits dynamic evolution. In the early stages, Prolactin activates STAT5a through JAK2, Its nuclear phosphorylation level is significantly correlated with the response rate to endocrine therapy and favorable prognosis (56); STAT5 and STAT3 mediate opposing regulatory effects on multiple key target genes; when both are simultaneously activated, STAT5 exerts a dominant role, thereby altering the gene expression profile and cellular phenotype driven by STAT3 (57). However, upon progression to endocrine resistance, aberrant activation of STAT3 signaling becomes a critical event, which not only mediates autonomous resistance through upregulation of genes such as BCL2 and MYC(Tumor cell-intrinsic effects) (58). Moreover, it actively reshapes the immunosuppressive microenvironment by inducing PD-L1 expression and recruiting immunosuppressive cells(Immunomodulatory effects on the tumor microenvironment), marking a critical transition of tumors from “hormone dependence” to “immunomodulatory dependence.” Collectively, these findings indicate that combining JAK-STAT pathway-targeted strategies with immune checkpoint inhibitors represents a promising approach to overcome immunotherapy resistance in breast cancer. Notably, the progressive loss of STAT5 signaling, rather than mere activation of STAT3, constitutes a key molecular event in endocrine resistance. Among these, the core positioning of JAK–STAT-targeted therapy lies in reversing the immunosuppressive microenvironment following acquired resistance to endocrine therapy, thereby creating the conditions for responsiveness to immune checkpoint inhibitors. Preclinical studies have confirmed that sustained activation of the STAT3 signaling axis is one of the key mechanisms mediating tamoxifen resistance, and that STAT3 inhibition can restore the sensitivity of resistant ER+ breast cancer models to endocrine therapy (59).

## 5 Molecular mechanisms of JAK-STAT pathway in promoting malignant progression of breast cancer

The core members of the JAK-STAT pathway, STAT3 and STAT5, play critical regulatory roles in breast cancer. In the breast cancer immune microenvironment, the two drive immunosuppression through distinct mechanisms: STAT3 upregulates PD-L1 and recruits MDSCs in tumor cells, whereas STAT5 maintains Treg function in T cells; ultimately, their effects converge in the same direction, both leading to the formation of an immunosuppressive microenvironment (50).

### 5.1 Promoting tumor cell proliferation and survival

STAT3/STAT5 drive cell cycle progression and metabolic reprogramming by upregulating genes such as Cyclin D1 and c-Myc, while inhibiting apoptosis through the activation of proteins including Bcl-2 and Bcl-xL, thereby facilitating rapid expansion of the tumor cell population (60). This process not only directly promotes tumor growth but also provides a sufficient cellular reservoir and heterogeneity basis for subsequent immune editing and evasion.

### 5.2 Enhancing invasion and metastatic capacity

STAT3 enhances tumor cell motility and invasive capacity by inducing epithelial-mesenchymal transition (EMT) and upregulating the expression of matrix metalloproteinases (MMP-2/9) (61). This enables tumor cells to evade local immune surveillance and disseminate to distant sites, where they establish new immunosuppressive metastatic lesions.

### 5.3 Maintaining cancer stem cell properties

STAT3 maintains the cancer stem cell pool endowed with self-renewal and multidrug resistance properties by directly activating stemness genes such as NANOG and OCT4 (octamer-binding transcription factor 4, also known as POU5F1), and by establishing an IL-6 autocrine loop (62).

### 5.4 Regulation of the tumor immune microenvironment

STAT3 serves as a central hub in this process. At the tumor cell level, STAT3 can directly bind to the promoter region of the PD-L1 (CD274) gene to drive its transcription; a strong association between p-STAT3 and PD-L1 expression has been observed in both breast cancer cell lines and patient samples, and pharmacological inhibition or siRNA silencing of STAT3 can significantly reduce PD-L1 expression; in addition, STAT3 can indirectly promote PD-L1 expression through transcription factors such as c-MYC. Notably, STAT1 and STAT3 can form heterodimers that jointly bind to the PD-L1 promoter; inhibition of STAT1 or STAT3 alone only partially downregulates PD-L1, whereas combined inhibition achieves complete suppression, confirming their functional synergy in PD-L1 regulation (63). At the myeloid cell level, the regulation of MDSCs by JAK/STAT signaling involves three biologically distinct processes: STAT3 promotes the recruitment of MDSCs to the tumor microenvironment by upregulating chemokines such as CCL2 (64); STAT3/STAT5 signaling promotes MDSC differentiation by downregulating IRF-8 expression, and IRF-8 levels in MDSCs from breast cancer patients decline as MDSC frequency increases (65); and sustained activation of STAT3 within MDSCs enhances their suppressive function by upregulating arginase 1 and ROS, thereby depleting amino acids essential for T cell proliferation and generating reactive oxygen species (66). At the lymphoid cell level, the regulation of Tregs by STAT5 likewise involves multiple steps: IL-2, IL-7, and IL-15 induce FOXP3 expression by activating STAT5, thereby driving the differentiation of naive CD4+T cells into Tregs (67); STAT5 maintains Treg survival and expansion through IL-2 signaling (68). Upon binding to PD-1 on the T cell surface, PD-L1 delivers inhibitory signals that lead to T cell functional exhaustion and diminished proliferative capacity (69). STAT3 inhibition can remodel the tumor immune microenvironment, manifesting as an increase in antitumor macrophage phenotypes and NK cell activation.

### 5.5 Promoting tumor angiogenesis

STAT3 directly upregulates the expression of vascular endothelial growth factor (VEGF), thereby promoting angiogenesis and influencing immune cell infiltration (70). In addition to VEGF regulation, JAK/STAT signaling also remodels tumor vascular function through multiple mechanisms: STAT3 upregulates CaMKIIδ via the IL-6/JAK/STAT3 axis to promote endothelial cell migration (71); the JAK2/STAT5 pathway maintains endothelial cell survival by inhibiting FAS/FASL-mediated apoptosis (72). At the level of immune cell trafficking, endothelial STAT3 activation promotes the infiltration of immunosuppressive cells and blocks the entry of antitumor immune cells, whereas endothelial STING-JAK1 signaling promotes vascular normalization and CD8+ T cell infiltration (73). Collectively, these mechanisms indicate that the JAK/STAT pathway not only drives angiogenesis through VEGF but also systematically shapes a pro-tumor vascular microenvironment by regulating vascular barrier function, endothelial cell survival and migration, and immune cell trafficking.

### 5.6 Implications of the functional antagonism between STAT1 and STAT3/STAT5 for targeted strategies

It should be emphasized that STAT3/STAT5-driven immunosuppression does not represent the entire picture of the JAK–STAT pathway in breast cancer. STAT1-mediated IFN signaling constitutes the core foundation of antitumor immune surveillance. Pan-JAK inhibitors or nonselective STAT inhibitors, while blocking STAT3/STAT5-driven pro-tumor signaling, also simultaneously suppress JAK1/STAT1-mediated IFN signaling, thereby impairing MHC class I antigen presentation and CXCL9/10 chemokine secretion (74). This attenuation of baseline antitumor immunity may even induce acquired resistance to immune checkpoint blockade (75). Therefore, targeted strategies should preferentially employ selective STAT3/STAT5 inhibition rather than pan-JAK family inhibition, thereby blocking immunosuppressive signaling while preserving STAT1-mediated immune surveillance.

On one hand, the JAK-STAT pathway drives tumor cell proliferation, survival, stemness maintenance, and invasion; on the other hand, it systematically reshapes the immunosuppressive microenvironment, characterized by elevated PD-L1 expression and infiltration of immunosuppressive cells. This dual regulation of both intrinsic tumor properties and the extrinsic microenvironment establishes the JAK-STAT pathway as a critical target for breast cancer immunotherapy.

## 6 JAK/STAT signaling pathway and breast cancer therapy

Given the critical role of the JAK-STAT pathway in regulating the breast cancer immune microenvironment, therapeutic strategies targeting this pathway have emerged as a significant research direction in combination immunotherapy.

### 6.1 Small-molecule JAK inhibitors

JAK inhibitors (JAKi) block STAT activation by competitively binding to the ATP-binding site. However, their kinase selectivity differs markedly: ruxolitinib exhibits substantially greater inhibitory activity against JAK1/2 than against JAK3, whereas fedratinib displays stronger inhibitory activity against JAK2 than against JAK1 (76). Preclinical studies have demonstrated that ruxolitinib inhibits tumor progression and modulates the immune microenvironment in TNBC models (77). However, early-phase clinical trials have demonstrated that JAK inhibitor monotherapy yields limited objective response rates (78). In metastatic TNBC, a phase II study of ruxolitinib monotherapy showed that none of the 21 evaluable patients achieved an objective response, with a median PFS of only 1.2 months; however, paired biopsies confirmed a significant decrease in the proportion of pSTAT3-positive cells, indicating that the drug did indeed hit its target. This disconnect between target inhibition and clinical benefit suggests that blocking JAK–STAT signaling alone is insufficient to dismantle the immunosuppressive network of TNBC (78). This limitation has deep mechanistic roots. On the one hand, following JAK–STAT inhibition, tumor cells can maintain survival through compensatory activation of parallel pathways such as PI3K/AKT and MAPK (79); on the other hand, pan-JAK inhibition, while blocking STAT3-driven pro-tumor signaling, also suppresses JAK1/STAT1-mediated IFN signaling, which is indispensable for MHC class I antigen presentation and CD8+ T cell activation (80). Ruxolitinib treatment leads to a reduction in intratumoral CD8+ T cells, further suggesting its immunosuppressive risk. Nevertheless, these findings provide a critical rationale for combination immunotherapy strategies.

### 6.2 Development of STAT inhibitors

Currently, strategies directly targeting STAT3 face technical challenges. SH2 domain inhibitors (e.g., OPB-51602) exert antitumor effects by preventing STAT dimerization; however, they are associated with toxicity concerns (81). The development dilemma of SH2 domain inhibitors stems from the intrinsic properties of their targets: the SH2 domain of STAT3 mediates membrane-proximal recruitment to the receptor and molecular dimerization by recognizing phosphotyrosine (pTyr) motifs; however, phosphotyrosine and its bioisosteres are highly susceptible to dephosphorylation by protein tyrosine phosphatases within cells, and their polyanionic nature severely impedes passive transmembrane diffusion, resulting in extremely low oral exposure (82). Although peptide-based inhibitors exhibit high binding affinity, they face challenges such as short plasma half-lives due to protease degradation and poor cell membrane permeability; non-peptide small-molecule inhibitors, while improving permeability and stability, do so at the expense of binding affinity (83). This tripartite trade-off among “high affinity, high permeability, and high stability” constitutes the core pharmacological obstacle that has thus far prevented any SH2 domain inhibitor from gaining FDA approval (15). Emerging proteolysis-targeting chimera (PROTAC) technology has shown promising prospects by inducing STAT protein degradation (84). In addition to SH2 domain inhibitors, strategies targeting the STAT3 DNA-binding domain and transactivation domain are also under exploration, yet both face challenges in selectivity and druggability. Currently, a decoy oligonucleotide strategy based on the DNA-binding domain has completed a phase 0 clinical trial in head and neck squamous cell carcinoma (85), whereas small-molecule inhibitors targeting the DNA-binding domain and transactivation domain inhibitors remain in the preclinical evaluation stage. PROTAC technology induces catalytic degradation of STAT3 protein via the ubiquitin–proteasome pathway by coupling a STAT3 ligand to an E3 ubiquitin ligase ligand, thereby obviating the need for sustained occupancy of the active site. SD-436 exhibits a DC_50_ of 10nM in SU-DHL-1 lymphoma cells, with negligible effects on STAT1 and STAT5 protein levels at concentrations up to 40μM, demonstrating 14–19-fold selectivity for STAT3 over STAT1 and STAT4; the oligonucleotide-conjugated C-STAT3D PROTAC likewise selectively degrades STAT3 without affecting STAT1 or STAT5 and displays TLR9^+^cell selectivity (86, 87). In preclinical models, a single intravenous dose of SD-436 at 5 mg/kg achieved rapid, complete, and durable STAT3 clearance in tumor tissue and induced complete and durable tumor regression in leukemia and lymphoma xenograft models under a weekly dosing regimen. It is noteworthy that STAT3 PROTACs have begun to enter the clinical validation stage. KT-333 has entered phase I clinical trials in hematologic malignancies and solid tumors, where target degradation and preliminary antitumor activity have been observed(NCT05225584); however, this trial did not specifically evaluate efficacy in breast cancer, and its efficacy and safety in breast cancer remain unexplored.

### 6.3 Upstream targeted intervention strategies

Targeting the IL-6/IL-6R axis represents an important strategy. The anti-IL-6R antibody tocilizumab has been shown to block IL-6/JAK/STAT3 signaling and influence the tumor immune microenvironment in preclinical studies (88). However, the efficacy of this approach depends on the degree of tumor dependence on IL-6 signaling. Notably, IL-6 plays physiological roles in T cell activation, B cell differentiation, and the acute-phase response (89), and systemic blockade may increase the risk of infection (90). To achieve tumor-specific targeting, researchers are exploring patient selection strategies based on IL-6R expression levels, aiming to block tumor pro-survival signaling while preserving normal immune surveillance function (91).

### 6.4 Combination therapeutic strategies

To overcome the limitations of monotherapy, combination therapeutic strategies have emerged as an important direction to enhance the efficacy of JAK-STAT pathway-targeted therapy. Current research has primarily focused on the following directions: chemotherapy-based combinations have demonstrated sensitization potential in TNBC, with mechanisms related to blocking STAT3-mediated apoptotic escape, although the relevant evidence remains at the preclinical stage (92). Endocrine-based combinations mainly target STAT3-driven resistance pathways in Luminal breast cancer and likewise rest primarily on preclinical evidence, having not yet been validated clinically (93). In contrast, anti-HER2 combinations have progressed further—a phase I/II clinical trial (NCT02066532) evaluated ruxolitinib combined with trastuzumab in HER2-positive resistant patients, but the median PFS was only 8.3 weeks, with efficacy falling short of expectations. In terms of combination with immunotherapy, JAK-STAT inhibitors modulate PD-L1 expression levels, reduce infiltration of immunosuppressive cells, and promote remodeling of the immune microenvironment, thereby creating favorable conditions for immune checkpoint inhibitor therapy (94). The theoretical basis of this strategy stems from the central role of the JAK-STAT signaling pathway in tumor immune evasion. Specifically, its mechanism of action can be understood at three levels. First, it directly regulates the expression of immune checkpoint molecules. The JAK-STAT signaling pathway serves as a central node in the transcriptional regulation of PD-L1. In various breast cancer subtypes, upon activation of the JAK-STAT axis by inflammatory cytokines such as IL-6 and IFN-γ, STAT transcription factors directly bind to the PD-L1 gene promoter to drive its expression. JAK inhibitors suppress JAK activity by blocking the ATP-binding site, thereby preventing STAT nuclear translocation and subsequently reducing PD-L1 levels to relieve T-cell suppression (24, 95). Second, they reshape immunosuppressive cell subsets. STAT3 directly controls the development and function of MDSCs and Tregs. When the JAK-STAT pathway is persistently activated, these two cell types accumulate abundantly in the tumor microenvironment and suppress immune responses through the secretion of factors such as TGF-β and IL-10 (96). JAK-STAT inhibitors can reduce the numbers of MDSCs and Tregs, attenuate their capacity to release suppressive factors, and restore the antitumor activity of effector T cells (97). Third, they promote the activation and infiltration of effector T cells. The hallmark of an immunosuppressive microenvironment is the ineffective infiltration or functional exhaustion of CD8+ T cells. By reducing PD-L1 levels and eliminating immunosuppressive cells such as MDSCs and Tregs, JAK-STAT inhibitors facilitate the entry of CD8+ T cells into the tumor and restore their cytotoxic function, thereby achieving a phenotypic shift of the tumor from “cold” to “hot” in terms of immune status (98). This shift is typically assessed in preclinical models by quantifying the ratio of tumor-infiltrating CD8+ T cells to Tregs (CD8+/Treg) via flow cytometry, as well as by measuring the expression levels of effector molecules such as IFN-γ and granzyme B in CD8+ T cells (99). However, this “cold-to-hot” conversion currently derives primarily from preclinical models, and clinical validation remains limited: the TBCRC 039 phase II trial showed that although ruxolitinib effectively inhibited pSTAT3, it simultaneously reduced GZMB+ T cells, suggesting that immunosuppressive effects may offset its antitumor activity. This remodeling process of the immune microenvironment provides a solid theoretical foundation for subsequent or concurrent administration of immune checkpoint inhibitors. It should be emphasized that this combination is not a simple “additive effect” but rather a mechanism-based synergy. Simple additivity refers to two drugs each acting independently, with efficacy merely equal to the sum of the individual agents; mechanism-based synergy, by contrast, refers to one drug altering the tumor microenvironment in a way that enables the other drug to achieve therapeutic effects that would otherwise be unattainable. Preclinical studies have provided direct evidence for this synergistic effect: in TNBC models, overexpression of PFDN5 (a co-chaperone protein regulating c-Myc) in combination with anti-PD-1 antibody synergistically suppresses tumor growth. Studies have shown that PFDN5 is downregulated in TNBC tissues, and its reduced expression is closely associated with poor patient prognosis. Mechanistically, upregulation of PFDN5 blocks the JAK2/STAT3/c-Myc pathway. The combination therapy enhances intratumoral infiltration of CD8+ T cells, thereby improving tumor control efficacy (100). Another study has revealed that targeting RAB10 (a small GTPase regulating vesicle trafficking) in combination with PD-L1 blockade also exerts synergistic antitumor effects. Mechanistically, breast cancer cells deliver RAB10 to macrophages via exosomes. Upon binding to IFNAR1 (the type I interferon receptor), RAB10 suppresses JAK1/STAT1 pathway phosphorylation, thereby impairing M1 macrophage polarization while promoting M2 polarization. This process upregulates the secretion of the immunosuppressive factor PD-L1 and reduces the activity of CD8+ T cells. In animal models, knockdown of RAB10 combined with anti-PD-L1 antibody treatment significantly synergistically inhibited tumor growth (101). Based on substantial preclinical evidence, related clinical trials are being actively pursued. For instance, a phase I trial (NCT03012230) conducted at the Mayo Clinic was the first to evaluate the safety and tolerability of ruxolitinib combined with pembrolizumab in metastatic TNBC. This study enrolled 12 patients with chemotherapy-refractory disease who received pembrolizumab (200 mg every 3 weeks) combined with ruxolitinib (10–20 mg BID). The results showed that no patient achieved an objective response (ORR 0%), and the median time to progression was 3 months (range 1.4–6.3 months); among the 6 patients at dose levels 0 and 1, 2 achieved 6-month disease stabilization. The principal toxicities were elevated transaminases (12/12), anemia/leukopenia (12/12), and fatigue (9/12), most of which were grade 1; 5 patients experienced grade ≥3 adverse events. The maximum tolerated dose was not reached (102). In addition to ruxolitinib, other JAK family inhibitors have also demonstrated synergistic effects when combined with immune checkpoint blockade. A study published in Anti-Cancer Drugs in 2025 was the first to demonstrate, in a humanized animal model of TNBC, that the selective TYK2 inhibitor deucravacitinib combined with the small-molecule PD-L1 inhibitor INCB086550 exerts significant synergistic antitumor activity (103). Furthermore, a phase II study (NCT06731153) is currently being conducted at Fudan University Shanghai Cancer Center, specifically evaluating the efficacy of the JAK1 inhibitor ivarmacitinib in combination with camrelizumab in patients with immunotherapy-resistant TNBC, offering a novel strategy to reverse immunotherapy resistance. This trial was initiated in December 2024 and is expected to be completed in 2027. It should be clarified that the JAK/STAT–ICB combination strategy is not applicable to all TNBC patients. TNBC exhibits substantial molecular heterogeneity, among which the immune-infiltrated subtype (BLIA) is more likely to benefit, whereas the immunosuppressive subtype (BLIS) and the mesenchymal subtype (MES/MLIA) may be primarily resistant to ICB (104). Therefore, identifying the patient population that would benefit requires reliable predictive biomarkers. However, no ideal biomarker currently exists—although STAT3 activation is associated with poor prognosis, this association falls within the realm of prognostic biomarkers and does not equate to its ability to predict response to JAK/STAT-targeted therapy (i.e., a predictive biomarker). PD-L1 immunohistochemistry likewise has limited reliability, as neither the KEYNOTE-522 nor the NeoTRIPaPDL1 trial effectively distinguished the benefiting population. More promising candidate biomarkers include: p-STAT1 positivity (associated with high PD-L1/HLA-I expression) (105); 9p24.1 amplification (encompassing JAK2/PD-L1, with an NGS-detected incidence of 5.1% in TNBC, far exceeding the 0.5% in non-TNBC) (106); immune cell infiltration signatures (such as the CTL-NK score) (107); and circulating biomarkers (such as PD-L1 expression on CTCs) (108). In addition, tumor heterogeneity and temporal changes in JAK/STAT activity should not be overlooked—a single biopsy may not accurately reflect differences in pathway activation status between primary and metastatic lesions. Therefore, patient selection should be based on molecular subtyping and dynamic biomarkers rather than on the general diagnosis of TNBC. However, the optimal dosing schedule, dosage determination, and patient selection strategies for combination therapy still require in-depth investigation, and careful attention must be paid to the potential overlapping risks of immune-related adverse events and JAK inhibitor-associated toxicities.

## 7 Summary and future perspectives

### 7.1 Summary

The JAK-STAT pathway plays a critical role in shaping the immune microenvironment and facilitating immune evasion in breast cancer, particularly in triple-negative breast cancer, by regulating PD-L1 expression, recruiting immunosuppressive cells, and attenuating effector immune cell function. Targeting this pathway in combination with immune checkpoint inhibitors offers a novel strategy to overcome immunotherapy resistance in breast cancer. Although preclinical evidence is substantial, a notable gap remains in clinical validation: objective response rates in existing clinical trials are limited, predictive biomarkers have yet to be established, and the optimal combination regimens are still being explored. Therefore, JAK–STAT-targeted therapy currently remains at a critical stage of translation from preclinical to clinical application, and its ultimate value still requires validation in larger-scale clinical trials.

### 7.2 Major challenges

The clinical translation of JAK-STAT pathway-targeted therapy faces multiple challenges. Major challenges include: compensatory resistance driven by pathway redundancy, impairment of JAK1/STAT1-mediated immune surveillance by pan-JAK inhibition, druggability bottlenecks of STAT inhibitors, and the lack of validated predictive biomarkers. (See Section 5 for details).

### 7.3 Future directions

Future research may focus on three directions: first, the development of highly selective inhibitors targeting specific JAK isoforms or STAT proteins to reduce immune-related adverse effects; second, given that conventional cell lines and immunodeficient xenograft models are limited in their ability to replicate the human tumor immune microenvironment, future studies should systematically integrate complementary systems such as syngeneic models, humanized mice, PDXs, and organoids, and enhance the reliability of translating preclinical findings to the clinic through cross-validation across multiple models; third, the exploration of precision combination therapeutic strategies based on immune biomarkers such as p-STAT and PD-L1; and fourth, in-depth elucidation of the dynamic mechanisms by which this pathway regulates the tumor immune microenvironment, thereby providing a theoretical foundation for optimizing combination immunotherapeutic regimens.
